# Phylogenetic analysis of serotype 19A-sequence type (ST)2331 Streptococcus pneumoniae associated with the Pneumococcal Molecular Epidemiology Network (PMEN)34 clone predominant in Japan after the introduction of the 7-valent pneumococcal conjugate vaccine (PCV7)

**DOI:** 10.1099/mgen.0.001808

**Published:** 2026-07-31

**Authors:** Shota Koide, Satoshi Nakano, Takao Fujisawa, Yutaka Ito, Bin Chang, Shigeru Suga, Yo Sugawara, Yukihiro Akeda, Motoyuki Sugai

**Affiliations:** 1 Antimicrobial Resistance Research Center, National Institute of Infectious Diseases, Japan Institute for Health Security, Tokyo, Japan; 2 National Hospital Organization, Mie National Hospital, Tsu, Japan; 3 Nagoya City University, Graduate School of Medical Science, Nagoya, Japan; 4 Department of Bacteriology I, National Institute of Infectious Diseases, Japan Institute for Health Security, Tokyo, Japan

**Keywords:** GPSC32, PMEN34, pneumococcal conjugate vaccine, serotype 19A, ST2331, *Streptococcus pneumoniae*, whole-genome sequencing

## Abstract

Following the introduction of the 7-valent pneumococcal conjugate vaccine (PCV7), serotype 19A *Streptococcus pneumoniae* emerged as a major cause of paediatric pneumococcal disease in Japan. Unlike global trends, in which multidrug-resistant serotype 19A-sequence type (ST)320 predominated, serotype 19A-ST2331 became one of the dominant lineages in Japan. The evolutionary origins of serotype 19A-ST2331 and the reasons for its domestic success remain unclear. To investigate the evolutionary origin and dissemination of the serotype 19A-ST2331 lineage, we analysed whole-genome sequences of 42 clonal complex (CC)2331 isolates collected in Japan between 2004 and 2017 and compared them with 825 genetically related global isolates. Phylogenetic analysis showed that Japanese serotype 19A-ST2331 isolates formed a distinct Japan-associated cluster within Global Pneumococcal Sequence Cluster 32 that was closely related to the globally disseminated Pneumococcal Molecular Epidemiology Network (PMEN)34 clone, typically represented by serotype 12F-ST218. Among the 42 Japanese CC2331 isolates, 40 were serotype 19A, whereas only two non-19A isolates were identified. Serotype 19A was absent from the 825 non-Japanese isolates, indicating that serotype 19A-ST2331 represents a globally rare, Japan-specific lineage. Most Japanese isolates remained susceptible to β-lactams, whereas 83.3% were erythromycin-resistant and carried either *mefA/E* or *ermB*. A highly clonal *mefA/E*-positive subclade was dated to a most recent common ancestor at ∼1998.5. These findings indicate that serotype 19A-ST2331 emerged before 2000 from an ST218-related ancestor through serotype switching and subsequently underwent local expansion in Japan. Although the ancestral intermediate was not represented in the present collection, the successful establishment of this clone may have been facilitated by the combined selective advantages conferred by the serotype 19A capsule and macrolide resistance in the post-PCV7 setting in Japan.

**Impact Statement**

Following the introduction of the 7-valent pneumococcal conjugate vaccine, *Streptococcus pneumoniae* serotype 19A emerged as a major cause of disease worldwide. In Japan, the serotype 19A-sequence type (ST)2331 lineage became predominant, yet its evolutionary origins and the reasons for its domestic success remained unclear. We used whole-genome sequencing to demonstrate that serotype 19A-ST2331 is a Japan-specific lineage derived from the globally disseminated Pneumococcal Molecular Epidemiology Network (PMEN)34 (serotype 12F-ST218) clone. We identified a highly clonal, macrolide-resistant subcluster that likely emerged in the late 1990s and spread nationwide and was characterized by remarkably low genetic diversity. Our findings indicate that the expansion of this clone was driven by the acquisition of the serotype 19A capsule and selective pressure from the historically high macrolide consumption in Japan. The present findings demonstrate that local antibiotic use and vaccination programmes can shape the evolution of specific bacterial pathogens, providing critical insights for global genomic surveillance into the emergence and regional establishment of vaccine-escape, multidrug-resistant lineages.

## Data Summary

The raw sequencing reads generated in this study are available in the DNA Data Bank of Japan (DDBJ; https://ddbj.nig.ac.jp/search) under the BioProject accession number PRJDB18569. The specific accession numbers analysed in this study, together with those for additional isolates obtained from public databases and previous studies, are listed in Table S1, available in the online version of this article.

## Introduction

*Streptococcus pneumoniae* is a leading cause of bacterial pneumonia, meningitis and sepsis in young children, with invasive pneumococcal disease (IPD) causing high morbidity and mortality [1]. To date, 108 serotypes have been identified based on structural differences in the capsular polysaccharide [2]. Pneumococcal conjugate vaccines (PCVs) targeting the capsular polysaccharide have been developed to prevent IPD. The 7-valent PCV (PCV7) was first introduced in the United States of America (USA) in 2000 and was subsequently implemented in many countries. In Japan, PCV7 was licensed in February 2010, initially administered on a voluntary basis and incorporated into the routine immunization programme in April 2013. PCV7 was replaced with PCV13 in 2013, whereas PCV15 and PCV20 became available in 2024.

Following the introduction of PCV7, the prevalence of non-PCV7 serotypes increased, particularly that of serotype 19A, which became one of the most frequently detected serotypes among paediatric IPD isolates and was often associated with multidrug resistance [3]. In many regions worldwide, 19A-sequence type (ST)320 emerged as one of the multidrug-resistant lineages after PCV7 implementation [4–8]. In Japan, nationwide surveillance of pneumococcal disease in paediatric patients from 2012 to 2017 was performed to monitor trends in seroprevalence and antimicrobial susceptibility [9, 10]. Serotype 19A was dominant in both IPD and non-IPD cases during the first study period (2012–2014) but declined markedly during the second period (2015–2017) following the introduction of PCV13 in 2013, which covers serotype 19A. In contrast to global trends, the dominant serotype 19A lineages in Japan were ST3111 and ST2331 rather than ST320, whereas the globally disseminated ST320 lineage was rarely detected [8]. Among these lineages, serotype 19A-ST3111 exhibited broader antimicrobial resistance than serotype 19A-ST2331 in nationwide surveillance analyses [9–11]. According to publicly available data, the ST3111 lineage has been detected predominantly in Japan, with limited identification in other countries [12–18]. Similarly, the ST2331 lineage has also been reported mainly in Japan, with only a small number of isolates identified outside the country [19, 20].

ST2331 is a triple-locus variant (TLV) of ST218, differing at the *gdh*, *gki* and *spi* loci in multilocus sequence typing (MLST). ST218 corresponds to the Pneumococcal Molecular Epidemiology Network (PMEN)34 (Denmark^12F^-34) lineage, as defined by the PMEN. PMEN34, typically represented by serotype 12F-ST218, spread successfully worldwide [21–25]. However, the evolutionary process through which this clone acquired serotype 19A, which increased after the introduction of PCV7 and is likely strongly associated with virulence and subsequently spread in Japan remains unclear. To address this gap, we obtained whole-genome sequences of CC2331 isolates detected in Japan during the last >10 years and performed phylogenetic and comparative genomic analyses with genetically closely related CC2331 isolates, including ST218 isolates, recovered from multiple countries.

## Methods

### Bacterial isolates

*S. pneumoniae* CC2331 isolates, defined as ST2331 and its single-locus variants (SLVs), double-locus variants and TLVs, collected in Japan between 2004 and 2017 were analysed from two independent collections.

The first collection comprised isolates obtained from medical institutions throughout Japan and was previously described as the YK collection [26] (see Supplementary Methods). These isolates were subsequently deposited in the Japan Antimicrobial Resistant Bacterial Bank (JARBB; https://jarbb.jp/en/about/), hereafter referred to as the JARBB collection. For the present study, 943 pneumococcal isolates derived from children aged ≤15 years were subjected to whole-genome sequencing (WGS), including 437 isolates collected between January and December 2004, 270 collected between January and December 2007 and 236 collected between January and December 2010. All isolates assigned to CC2331 based on *in silico* MLST results were included in subsequent analyses.

The second collection comprised 1,358 isolates obtained between January 2012 and December 2017 through the Pneumocatch nationwide surveillance study in Japan [9, 10], hereafter referred to as the Pneumocatch collection. This study enrolled 823 and 509 paediatric patients with IPD and non-IPD, respectively, from whom 841 and 517 isolates were obtained. All CC2331 isolates were selected based on the MLST profiles reported in these studies [9, 10]. WGS data were obtained from BioProject PRJDB14360, where available and the remaining isolates were sequenced in the present study.

### Bacterial genome sequence data of international isolates

Whole-genome sequence data of *S. pneumoniae* CC2331 isolates from outside Japan were downloaded and analysed together with isolate data from Japan.

To identify candidate global isolates, a systematic search was performed in June 2025 across public pneumococcal genomic resources, including the PubMLST *S. pneumoniae* isolate database (https://pubmlst.org/bigsdb?db=pubmlst_spneumoniae_isolates), the Monocle Data Viewer of the Global Pneumococcal Sequencing project (https://data-viewer.monocle.sanger.ac.uk/project/gps) and Pathogenwatch (https://pathogen.watch), as well as PubMed search using the terms ‘*Streptococcus pneumoniae*’ AND (‘whole-genome sequencing’ OR WGS), restricted to English-language publications. Studies were included when per-isolate ST, country of origin, year of isolation and raw-read accession numbers were available. For isolates belonging to CC2331, metadata and genome sequence data were downloaded from public sequence archives.

### Antimicrobial susceptibility testing

Isolates from the JARBB and Pneumocatch collections were subjected to antimicrobial susceptibility testing using the microdilution method following the Clinical and Laboratory Standards Institute (CLSI) guidelines [27]. Minimum inhibitory concentrations were determined for penicillin (PEN), cefotaxime (CTX), meropenem (MEM) and erythromycin (ERY) according to the 2024 CLSI standards. Susceptibility categories were defined as follows: PEN-susceptible (PEN-S, ≤0.06 mg l^−1^) and PEN-resistant (PEN-R, >0.06 mg l^−1^); CTX-susceptible (CTX-S, ≤0.5 mg l^−1^) and CTX-nonsusceptible (CTX-NS, ≥1.0 mg l^−1^); MEM-susceptible (MEM-S, ≤0.25 mg l^−1^) and MEM-nonsusceptible (MEM-NS, ≥0.5 mg l^−1^); and ERY-susceptible (ERY-S, ≤0.25 mg l^−1^), ERY-intermediate (ERY-I, 0.5 mg l^−1^) and ERY-resistant (ERY-R, ≥1.0 mg l^−1^).

### Whole-genome sequencing

Genomic DNA from isolates in the JARBB and Pneumocatch collections was extracted using the QIAamp DNA Mini Kit (QIAGEN, Hilden, Germany) according to the manufacturer’s instructions. DNA libraries were prepared using the Enzymatics 5×WGS fragmentation mix and WGS ligase reagents (QIAGEN). Paired-end sequencing (2×150 bp) was performed on the Illumina HiSeq X Five platform (Macrogen Japan Corporation, Tokyo, Japan).

### Bioinformatics

#### Assembly and quality assessment

After raw-read trimming using fastp v0.23.4 [28], the reads were assembled using Shovill v1.1.0 [29]. Basic statistics for the assembled genomes were computed using QUAST v5.2.0 [30], and contamination rates were calculated using CheckM2 v1.0.2 [31]. Samples with an N50 value <10,000 or a contamination rate ≥1.0 were excluded from the analysis.

#### MLST, serotyping and global pneumococcal sequence cluster assignment

Isolates that passed quality filtering were subjected to *in silico* MLST [32] using mlst v2.23.0 [33, 34]. Isolates from Japan were also serotyped using pneumococcal typing antisera (Statens Serum Institut, Copenhagen, Denmark), whereas global isolates were serotyped using SeroBA v1.0.2 [35]. Global pneumococcal sequence clusters (GPSCs) were assigned using the GPSC assignment module in Pathogenwatch (v2.0.1-gpsc-v8) [36].

#### Penicillin-binding protein typing and MIC prediction

Penicillin-binding protein (PBP)1a, PBP2b and PBP2x typing was performed using the SPN-PBP-AMR module implemented in Pathogenwatch (spn_pbp_amr v0.0.1). When a PBP type was designated as ‘NEW,’ isolates were further analysed locally using two reference databases: SPN_Reference_DB [37, 38] and a Japanese PBP database containing previously reported types [8, 11].

#### Detection of antimicrobial resistance factors and pili genes

The presence or absence of *ermB*, *mefA/E*, *tetM*, *tetO, rrgA-1* (pili-1) and *pitB-1* (pili-2) was assessed using an in-house genome analysis pipeline [39]. Amino acid substitutions in GyrA, ParC and FolA, as well as *folP* insertions, were also analysed. For isolates with macrolide resistance genes (*ermB* or *mefA/E*) and *tetM*, the structure of the genomic region carrying these genes was determined based on the Tn*916* sequence (GenBank accession U09422.1).

### Phylogenetic analysis and Bayesian dating

A maximum likelihood phylogenetic tree was constructed using kSNP4 v4.1 [40] for all CC2331 isolates from Japan and other regions. Subsequently, to generate a higher-resolution phylogeny for the cluster comprising Japanese isolates, a maximum likelihood phylogenetic tree was constructed after removing recombination regions identified using Gubbins v3.3.1 [41]. Average pairwise SNPs were calculated using snp-dists v0.8.2 [42]. Bayesian molecular dating of nodes from the Gubbins output tree was performed using BEAST X v10.5.0 [43]. All phylogenetic trees were visualized together with metadata using iTOL [44].

### Statistical analysis

Statistical analysis was performed using SciPy v1.13.0 [45]. Categorical variables were compared using Pearson’s chi-square test on 2×2 contingency tables. All tests were two-sided, and a *P*-value<0.05 was considered statistically significant.

## Results

### Bacterial isolates and genome data

A total of 42 CC2331 isolates from Japan were included in the present study, comprising 11 isolates selected from the 943-isolate JARBB collection and 31 isolates from the Pneumocatch collection. Among the 11 CC2331 isolates from the JARBB collection, 9 were collected in 2004 and 2 in 2007. Among the 31 isolates from the Pneumocatch collection, 19 were collected between 2012 and 2014 and the remaining 12 were collected between 2015 and 2017. Geographically, these 42 isolates from Japan were recovered from 18 of the 47 prefectures in Japan, ranging from 1 to 11 isolates per prefecture (median, 2 isolates) (Fig. S1).

In total, 825 global isolates within the TLV threshold of ST2331 were included and were analysed together with the Japanese isolates. These 825 isolates originated from 26 countries, of which 56.1% (*n*=463) were from the USA, 14.5% (*n*=120) from Kenya and 6.8% (*n*=56) from the United Kingdom. These isolates were collected between 1988 and 2020, including 33 isolates collected between 1988 and 2000, 208 between 2001 and 2010 and 584 between 2011 and 2020.

All 867 isolates are listed in Table S1, together with accession number, PMID, reference (author and publication year or database name and URL), country of origin and year of isolation. In addition, for the Japanese isolates, other available metadata, including the month of isolation, patient age, specimen source, IPD classification and geographic region, are also provided.

### Whole-genome sequencing statistics

For the 42 CC2331 isolates from Japan analysed here, the mean±sd number of contigs was 86±57, the N50 value (defined as the shortest contig length required to cover 50% of the genome) was 78,265 bp ±28,820 bp and the mapping depth was 194×±142×. Statistics for all 867 genomes, including the 825 downloaded genomes from outside Japan, are provided in Table S1.

### Serotype and genetic lineages

With the exception of 2 isolates, all 42 CC2331 isolates from Japan belonged to serotype 19A. One isolate, serotype 12F-ST18693 (a TLV of ST2331 and an SLV of ST218), was identified in 2004, whereas a second isolate, serotype 9N-ST405 (also a TLV of ST2331 and an SLV of ST218), was detected in 2019. Of the 40 serotype 19A isolates from Japan, 34 were ST2331, whereas the remaining 6 were SLVs of ST2331. Globally, CC2331 isolates comprised 16 different serotypes, and when Japanese isolates were included, the entire CC2331-related dataset comprised 17 serotypes because serotype 19A was observed exclusively among Japanese ST2331 and SLV isolates. The serotype 12F-ST18693 lineage was specific to Japan, whereas serotype 9N-ST405 was detected across six different regions. Of the 867 isolates analysed in the present study, 850 were assigned to 1 of 3 GPSCs: GPSC32 (*n*=702, 81.2%; Fig. S2a), GPSC233 (*n*=113, 13.1%; Fig. S2b) and GPSC61 (*n*=35, 4.0%; Fig. S2c). The remaining 17 isolates (2.0%) were not assigned to any GPSC, including 6 isolates from Japan (serotype 19A-ST2331) (Fig. S2d).

Among the global isolates, five ST2331 isolates were identified: three serotype 7F isolates (two from the Netherlands and one from the USA) and two serotype eight isolates (both from Russia). ST2331 and ST218 (PMEN34), both of which belonged to GPSC32, differed by three MLST alleles (i.e. TLVs). Among the 248 ST218 isolates, four serotypes were identified: 12F (*n*=231, 93.1%), 7F (*n*=15, 6.0%) and one isolate each of serotypes 12B and 3 (0.4% each).

### Antimicrobial susceptibility, resistance genes, PBP type and pili genes

We performed antimicrobial susceptibility testing for all 42 CC2331 isolates from Japan. Resistance to β-lactams was infrequent: PEN-R (2.4%), CTX-NS (7.1%) and MEM-NS (0%) (Table 1). In contrast, 35 isolates (83.3%) were ERY-R and harboured a macrolide resistance gene, either *mefA/E* (*n*=29, 82.9%) or *ermB* (*n*=6, 17.1%). Among the 825 non-Japanese isolates, macrolide resistance genes were detected less frequently: *mefA/E* was identified in 121 isolates (14.7%) from North America, whereas *ermB* was not detected. All *mefA/E*-positive global isolates belonged to GPSC32. The prevalence of macrolide resistance genes was significantly higher among Japanese isolates than among non-Japanese isolates (chi-square test, *P*<0.001; Table 2).

**Table 1.** Antimicrobial susceptibilities of serotype 19 A-CC2331 isolates from Japan, 2004–2017

Antibiotics	No. of isolates (%)	
	Susceptible	Nonsusceptible^*^
Penicillin	41 (97.6)	1 (2.4)
Cefotaxime	39 (92.9%)	3 (7.1%)
Meropenem	42 (100)	0 (0)
Erythromycin	7 (16.7)	35 (83.3)

*Nonsusceptibility included both intermediate resistance and resistance. Penicillin and cefotaxime susceptibility were interpreted according to the meningitis breakpoints.

**Table 2.** Prevalence of macrolide resistance genes in CC2331 isolates overall and within GPSC32 by region

*ermB* or *mefA/E*	Overall (*n*=867)			GPSC32 (*n*=702)		
	Region		*P*-value	Region		*P*-value
	Japan	Others		Japan	Others	
Yes	35	121	<0.001	31	121	<0.001
No	7	704		5	545	

**Fig. 1.**
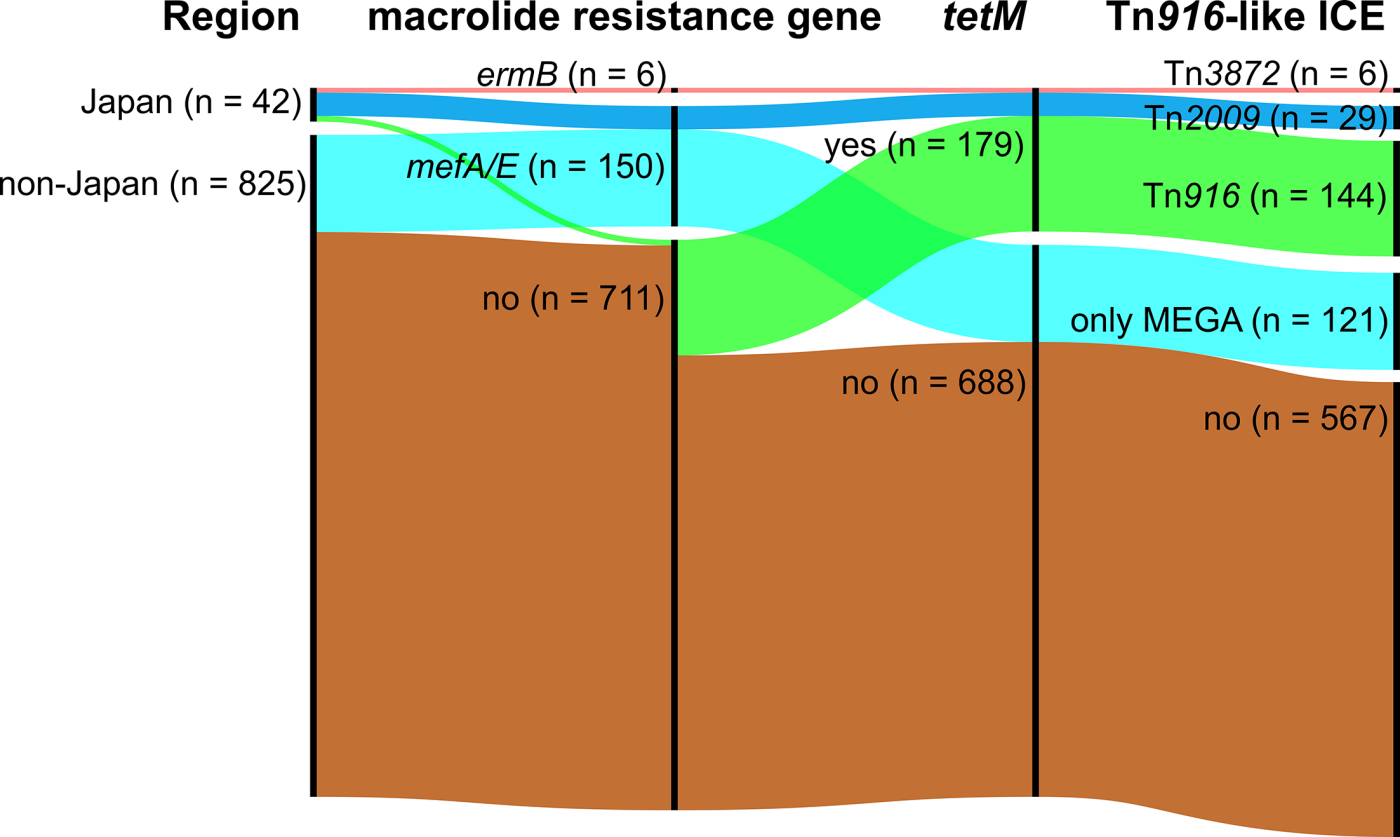
Sankey diagram illustrating the distribution of macrolide resistance genes and Tn*916*-like ICEs among CC2331 isolates. Tn*3872* carrying *ermB* (*n*=6) and Tn*2009* carrying *mefA/E* (*n*=29) were identified exclusively in isolates from Japan. All *mefA/E* genes carried by global isolates were located on the *mefE*-carrying genetic element (mega). Tn*916* without macrolide resistance genes was identified in 144 isolates, including 139 global isolates and 5 isolates from Japan.

We also investigated Tn*916*-like integrative and conjugative elements (ICEs). Tn*3872* was detected in all *ermB*-positive isolates (Fig. 1). All *mefA/E* genes in Japanese isolates were carried by Tn*2009*, whereas those in global isolates were carried by the *mefE*-carrying genetic element (mega) [46] without Tn*916*-like ICEs. Tn*916* was identified in 144 isolates overall, including 5 isolates from Japan.

None of the 867 isolates harboured *rrgA-1* (pili-1). In contrast, 32 isolates (3.7%) carried *pitB-1* (pili-2), all of which belonged to GPSC32 and were serotype 7F.

The most prevalent PBP profile (PBP1a_PBP2b_PBP2x) among CC2331 isolates from Japan was 2_0_689 (25/42, 59.5%), followed by 2_0_137 (7/42, 16.7%). One isolate carried a novel PBP2x type (see Supplementary Results).

### Phylogenetic analysis

**Fig. 2.**
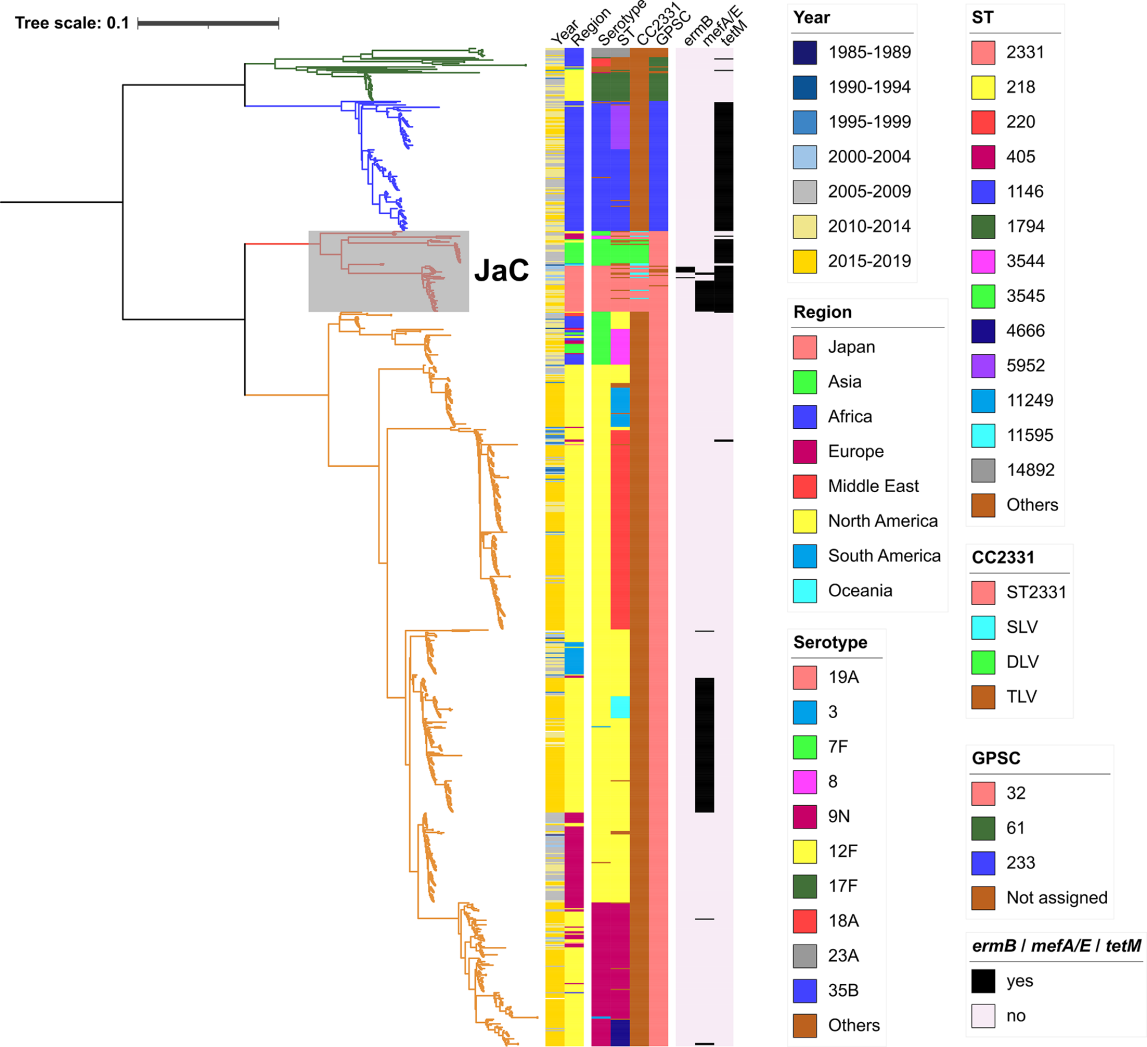
Maximum-likelihood phylogenetic tree of 867 clonal complex (CC)2331 isolates constructed using kSNP4. The tree was midpoint-rooted during visualization in iTOL. Four distinct subclusters were identified: isolates assigned to GPSC61 (46 isolates; green branches) and GPSC233 (113 isolates; blue branches) and two subclusters within GPSC32 comprising 70 ST2331 isolates (red branches) and 638 ST218 isolates (yellow branches). The ST2331 subcluster was designated the JaC(grey shaded). Coloured annotation strips indicate year of isolation, region of isolation, serotype, ST, CC2331 subgroup, GPSC and the presence of Tn*916*-like ICEs-associated resistance genes (*ermB*, *mefA/E* and *tetM*).

In the maximum-likelihood phylogenetic tree of 867 CC2331 isolates constructed using kSNP4, two major clades were identified (Fig. 2). One clade comprised isolates assigned to GPSC61 (46 isolates; green branches) and GPSC233 (113 isolates; blue branches). The other clade corresponded to GPSC32 (708 isolates) and was further divided into 2 subclades: a subclade containing ST2331 isolates (70 isolates; red branches) and a subclade containing ST218 isolates (638 isolates; yellow branches). The ST2331 subclade included 40 isolates from Japan (57.1%) and was designated the Japan-associated cluster (JaC; grey shaded). Of the 42 Japanese isolates, 40 were located within the JaC, whereas the remaining 2 isolates (serotypes 9N and 12F) were positioned within the ST218 subclade. Compared with the other clades, the ST218 subclade exhibited greater diversity, encompassing isolates from 22 countries and 5 serotypes.

### Japan-associated cluster

**Fig. 3.**
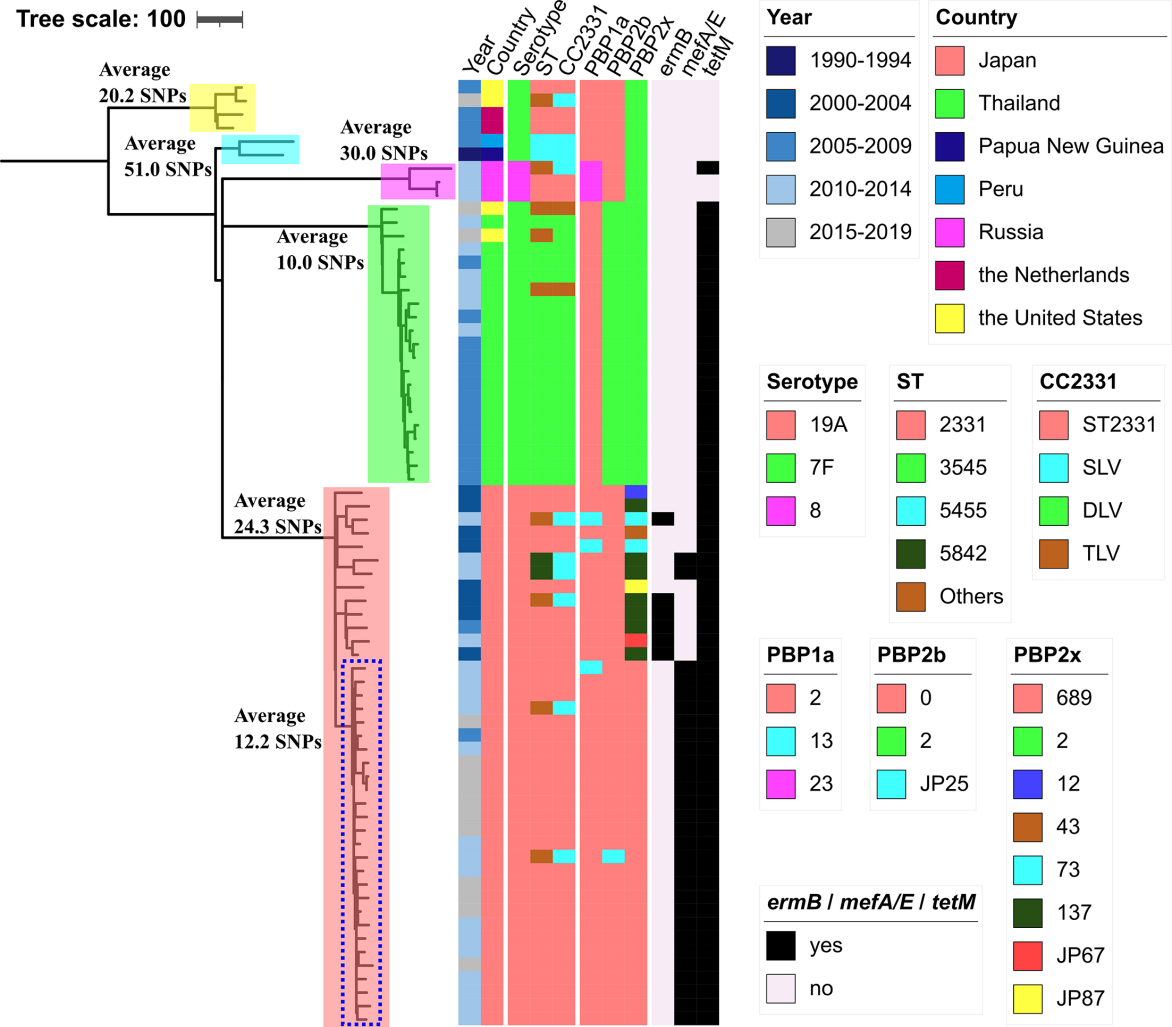
Recombination-censored maximum-likelihood phylogenetic tree of the JaC isolates, including all ST2331 isolates, reconstructed using Gubbins. The tree was midpoint-rooted during visualization in iTOL. Five subclades were identified. The serotype 7F-ST3545 clade exhibited the lowest average pairwise SNP distance (10.0 SNPs), followed by a *mefA/E*-positive subclade within the serotype 19A isolates (enclosed by a blue dashed line), with the second-lowest average pairwise SNP distance (12.2 SNPs). The average pairwise SNP distance between these two major subclades was 147.2 SNPs. Coloured annotation strips indicate year of isolation, country of isolation, serotype, ST, CC2331 subgroup, PBP1a, PBP2b and PBP2x types and the presence of Tn*916*-like ICEs-associated resistance genes (*ermB*, *mefA/E* and *tetM*).

To further investigate the genetic structure of the JaC, we reconstructed a recombination-censored maximum-likelihood phylogeny using Gubbins, which revealed five subclades (Fig. 3). Two of the five subclades were major clusters: one consisted predominantly of serotype 7F-ST3545 isolates, whereas the other comprised serotype 19A-ST2331 isolates from Japan. The serotype 19A clade had an average pairwise SNP distance of 24.3, whereas the *mefA/E*-positive subclade among serotype 19A isolates, enclosed by the blue dashed line in Fig. 3, exhibited a lower average pairwise SNP distance of 12.2. The average pairwise SNP distance between the serotype 7F-ST3545 clade isolates (*n*=21) and the *mefA/E*-positive subclade isolates from Japan (*n*=27) was 147.2 SNPs. PBP profiles further distinguished the JaC subclades (Fig. 3). PBP1a type was two in most isolates, with the exception of all Russian serotype eight isolates (PBP1a type 23) and three Japanese isolates (PBP1a type 13). PBP2b type was two in all isolates within the serotype 7F-ST3545 clade and type 0 in all remaining isolates. PBP2x type was two in all non-Japanese isolates, whereas Japanese isolates carried predominantly PBP2x type 689 (*n*=27), followed by PBP2x type 137 (*n*=7), with several additional types also identified. Focusing exclusively on the ST2331 isolates, three clusters were identified. The first cluster included three serotype 7F isolates, two recovered in the Netherlands and one in the USA. The second cluster comprised serotype eight isolates, both recovered in Russia. The third cluster contained 34 isolates from Japan, all of which were serotype 19A.

### Dating the serotype 19A-ST2331 subclade in Japan

**Fig. 4.**
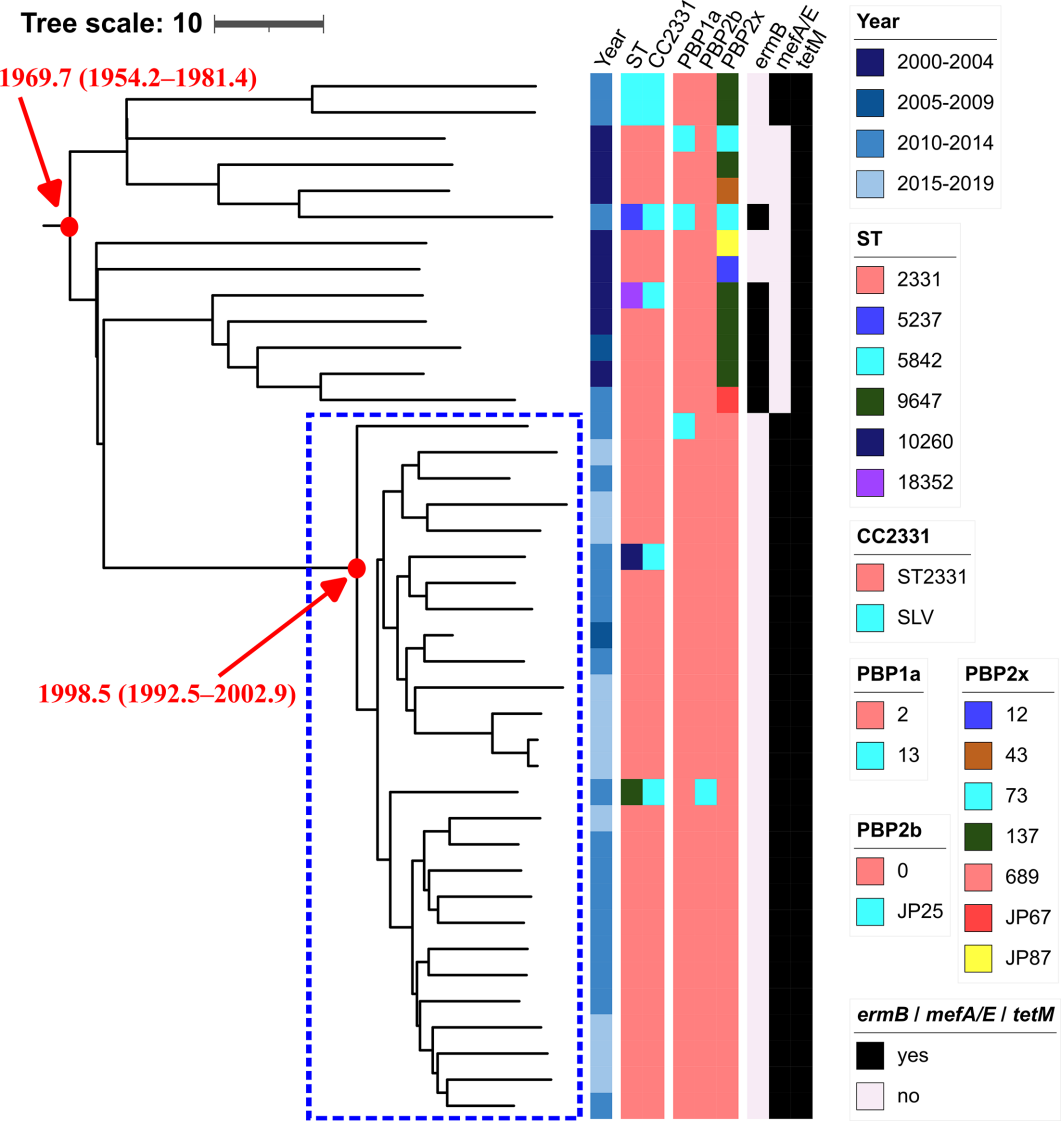
Recombination-censored, time-calibrated phylogenetic tree inferred using BEAST X. The tree includes all 40 serotype 19A isolates from Japan. The subcluster enclosed by a blue dashed line corresponds to the same *mefA/E*-positive subclade highlighted in Fig. 3. Coloured annotation strips indicate year of isolation, ST, CC2331 subgroup, PBP1a, PBP2b and PBP2x types and Tn*916*-like ICEs-associated resistance genes (*ermB*, *mefA/E* and *tetM*).

The root of the dataset was dated to 1969.7 (95% highest posterior density [HPD]: 1954.2–1981.4) (Fig. 4). The node corresponding to the *mefA/E*-positive subclade (*n*=27) was dated to 1998.5 (95% HPD: 1992.5–2002.9). The estimated median substitution rate was 9.59×10⁻⁷ substitutions/site/year (95% HPD: 6.53×10^−7^–1.27×10^−6^). Supplementary analyses supporting the molecular dating approach are provided in the corresponding ‘Dating the serotype 19A-ST2331 subclade in Japan’ section in the Supplementary Results.

## Discussion

To clarify the phylogenetic placement of the serotype 19A-ST2331 clone, which became prevalent in Japan after the introduction of PCV7, within the global pneumococcal population and to investigate the mechanisms underlying its establishment in Japan, we obtained whole-genome sequence data from 42 CC2331 isolates collected in Japan between 2004 and 2017 and performed comparative genomic analyses with 825 closely related isolates from other countries. A key finding was that ST2331 is a TLV of ST218, a globally disseminated clone designated PMEN34. As PMEN34 is represented by the Denmark^12F^-34 clone, serotype 12F is the serotype most commonly associated with the ST218 lineage. This clone was first isolated in Denmark in 1988 [47] and was detected mainly in IPD cases, including several outbreak events [22, 23, 48, 49]. Although serotype 12F-ST218 has been described in numerous epidemiological studies, comprehensive genomic analyses of its closely related variants remain limited.

In the present study, we performed a comprehensive whole-genome comparative analysis encompassing ST2331 and its TLVs. Our findings demonstrated that serotype 19A-ST2331 represents a Japan-specific lineage. Despite being assigned to the same GPSC32 as serotype 12F-ST218 (PMEN34), phylogenetic analysis clearly showed that serotype 19A-ST2331 formed a distinct cluster separate from serotype 12F-ST218. The most closely related clone with the largest number of available isolates was serotype 7F-ST3545 detected in Thailand. However, because these Thailand isolates were obtained through a 2 year longitudinal sampling study of mothers and infants in the highly populated Maela refugee camp [50], multiple isolates derived from the same individual may have been included. Consistent with this phylogenetic distinction, the average pairwise SNP distance between the Japanese *mefA/E*-positive serotype 19A-ST2331 subclade and the serotype 7F-ST3545 Thailand subclade was 147.2 SNPs. Thus, our analysis did not identify any major clone that could represent a clear genetic intermediate between the serotype 19A-ST2331 lineage that spread in Japan and the globally disseminated serotype 12F-ST218 lineage. This level of divergence argues against recent direct international transmission and instead supports local evolution and expansion of this lineage in Japan after divergence from other ST2331-related lineages.

Despite these limitations, one of the few available clues regarding the ancestor of serotype 19A-ST2331 was a serotype 12F-ST18693 isolate detected in Japan in 2004. In the phylogenetic tree, this isolate, which was a TLV of ST2331, clustered within the serotype 12F-ST220 clade, a SLV of ST218. Given the genetic distance from ST2331, whether the ancestor of serotype 12F-ST18693 was directly ancestral to serotype 19A-ST2331 could not be determined. Nevertheless, its detection in the early 2000s raises the possibility that a lineage more closely related to serotype 12F-ST218 than to serotype 19A-ST2331, and potentially ancestral to the latter, had already been introduced into Japan before the emergence of ST2331.

Among the 867 isolates analysed in the present study, which comprised 17 serotypes in total, the only isolates assigned to serotype 19A were ST2331 and its SLV isolates detected in Japan. The acquisition of serotype 19A through genetic recombination was likely a stochastic event. However, the subsequent spread of this lineage in Japan after the acquisition of serotype 19A may have been attributable to characteristics inherent to the serotype 19A capsule. Weinberger *et al.* [51] reported that serotype 19A was associated with a lower metabolic cost and, consistent with their model, may also have been associated with a thicker capsule and increased resistance to neutrophil-mediated killing. In addition, Chun *et al.* [52] reported that the serotype 19A capsule facilitates attachment to airway epithelial cells.

Among the serotype 19A-ST2331 isolates that circulated in Japan, we identified a *mefA/E*-positive subcluster. This subcluster represented a highly clonal group of isolates, with an average pairwise SNP distance of 12.2. This value is remarkably low, particularly given that the average pairwise SNP distance among isolates collected in the Maela camp described above was 10.0. Approximately all isolates in this subcluster (26/27) were derived from the Pneumocatch collection, a nationwide surveillance-based collection, providing strong support for the high transmissibility of this subcluster. Furthermore, isolates in this cluster were resistant to macrolide antibiotics because of the presence of *mefA/E*. This resistance may have contributed to the spread of this lineage in Japan. Japan has a higher proportion of macrolide antibiotics in total antibiotic consumption than several other countries in the European Union [53]. Although quantitative nationwide antimicrobial consumption data in Japan for the period before the early 2000s, corresponding to the estimated divergence period of this resistant clone, remain limited, macrolide antibiotics have been used extensively in Japan since the 1990s [54–56]. Given that macrolide consumption has been associated with increased macrolide resistance in pneumococci [57], the macrolide-resistant serotype 19 A-CC2331 lineage may have expanded under such selective pressure.

Taken together, these findings suggest that dissemination of the *mefA/E*-positive serotype 19A-ST2331 subclade within Japan was associated with the acquisition of both the serotype 19A capsule and macrolide resistance. Similar patterns have been reported in which regional dissemination of specific pneumococcal clones was associated with serotype switching and antimicrobial resistance acquisition. Serotype 35B-ST156 emerged after PCV13 introduction through serotype switching and acquisition of multidrug resistance. Previous studies from the USA suggest that this lineage arose through serotype switching between a penicillin-resistant serotype 35B-ST558 lineage and serotype 9V-ST156 [58, 59]. Transmission and dissemination of the same clone have also been reported in Japan [60]. In addition, serotype 24F is a non-vaccine serotype not included in PCV20, and increases in its prevalence have been reported in multiple countries in the context of serotype replacement following PCV introduction. Lo *et al.* demonstrated that the post-PCV13 increase in serotype 24F in France was primarily attributable to the expansion of the multidrug-resistant GPSC10 (CC230) clone, which exhibits resistance to penicillin, cotrimoxazole, erythromycin and tetracycline. The same study further reported that a serotype shift within GPSC10 from 19A to 24F was also observed in Spain after PCV13 introduction. In addition, the authors suggested that antimicrobial selective pressure, in addition to vaccine selective pressure, may have contributed to GPSC10-24F expansion [61]. Because serotype 19A is included in PCV13, serotype 19 A-CC2331 was not detected among paediatric IPD isolates collected in Japan during 2020–2023 [62]. Moreover, no CC2331 isolates with other serotypes were identified during this period. However, this finding should be interpreted with caution because the number of detected IPD cases was low during this period, partly because of the COVID-19 pandemic [63] and rare emerging variants may, therefore, have remained undetected. Given that the serotype 12F-ST218 lineage appears to represent an internationally disseminated clone with high epidemic potential, continued genomic surveillance is warranted not only for clones related to the rapidly expanding 19 A-CC2331 lineage in Japan but also for 12F-ST218-related lineages that may acquire nonvaccine serotypes or antimicrobial resistance through serotype switching and other genetic changes.

The present study had several limitations. We were unable to identify any isolates in public databases or in our collections that were genetically intermediate between ST218 and ST2331. Consequently, we could not infer the direct ancestral strain of serotype 19A-ST2331. Because no large-scale pneumococcal collection predating the YK collection exists in Japan, obtaining further information regarding this issue will likely remain impossible.

In conclusion, we demonstrated that the serotype 19A-ST2331 (CC2331) lineage in Japan represents a globally rare PMEN34-related clone. In particular, the *mefA/E*-positive serotype 19A-ST2331 isolates identified in Japan after the introduction of PCV7 showed a remarkably low average pairwise SNP distance, suggesting that this clone has high transmissibility and invasiveness. The success of this clone may have been influenced by the serotype 19A capsule and selective pressure associated with the extensive use of macrolides in Japan.

## Supplementary material

10.1099/mgen.0.001808 Supplementary Material 1.

10.1099/mgen.0.001808 Table S1.

## Abbreviations

CC: clonal complex
CTX: cefotaxime
DLV: double-locus variant
ERY: erythromycin
GPSC: global pneumococcal sequence cluster
ICE: integrative and conjugative element
IPD: invasive pneumococcal disease
JaC: Japan-associated cluster
JARBB: Japan Antimicrobial Resistant Bacterial Bank
MEM: meropenem
MLST: multilocus sequence typing
PBP: penicillin-binding protein
PCV7: 7-valent pneumococcal conjugate vaccine
PCV: pneumococcal conjugate vaccine
PEN: penicillin
PMEN: Pneumococcal Molecular Epidemiology Network
SLV: single-locus variant
ST: sequence type
TLV: triple-locus variant
WGS: whole-genome sequencing
