# Supplementary material for: Phylogenetic analysis of serotype 19A-sequence type (ST)2331 Streptococcus pneumoniae associated with the Pneumococcal Molecular Epidemiology Network (PMEN)34 clone predominant in Japan after the introduction of the 7-valent pneumococcal conjugate vaccine (PCV7)
Source: Microb Genom. 2026 Jul 31;12(7):001808. doi: 10.1099/mgen.0.001808 (PMC13426510; doi:10.1099/mgen.0.001808)
Supplement: Supplementary Material 1. [file mgen-12-01808-s001.pdf]

Phylogenetic analysis of serotype 19A-ST2331 *Streptococcus pneumoniae* associated with the PMEN34 clone predominant in Japan after PCV7 introduction

Supplementary Methods

Bacterial isolates: the original study of the YK collection

The YK collection is a nationwide collection of clinical isolates established under the framework of postmarketing surveillance regulated by the Japanese Ministry of Health, Labour and Welfare, with the aim of monitoring antimicrobial susceptibility trends following the domestic launch of levofloxacin in 1994 [1]. The collection was built through a nationwide surveillance program conducted biennially or triennially at 10 time points between 1994 and 2016. The number of participating institutions varied across time points (24–77 per time point), with a total of 100 distinct medical centers contributing isolates over the course of the surveillance. This collection included the following bacterial species: *Staphylococcus aureus* (methicillin-susceptible and methicillin-resistant), *Streptococcus pneumoniae*, *Streptococcus pyogenes*, *Escherichia coli*, *Klebsiella pneumoniae*, *Salmonella* spp., *Pseudomonas aeruginosa* isolated from urinary tract infections (UTIs) or respiratory tract infections (RTIs), *Haemophilus influenzae*, and *Acinetobacter* spp. In total, 54,933 isolates were collected, of which 5,372 were *S. pneumoniae*. After initial identification at each participating center, isolates were sent to a central laboratory (BML, Inc., Saitama, Japan) for reidentification and antimicrobial susceptibility testing using the broth microdilution method according to the Clinical and Laboratory Standards Institute (CLSI) guidelines, with interpretation based on CLSI M100-S27 or EUCAST version 7.1 breakpoints. The collection was subsequently deposited in the Japan Antimicrobial Resistant Bacterial Bank (JARBB) (https://jarbb.jp/en/about/) and is hereafter referred to as the JARBB collection.

Bacterial isolates: YK collection isolates used in this study

A total of 5,372 *S. pneumoniae* isolates are included in the YK collection across the entire surveillance period (ranging from 253 to 1,010 isolates per time point). In the present study, to align with the Pneumocatch collection [2, 3], which targeted pediatric patients, isolates from the JARBB collection were similarly restricted to those obtained from pediatric patients aged ≤15 years. Considering the study period of the Pneumocatch surveillance study (2012–2017), all isolates from

2004, 2007, and 2010 that were recoverable from frozen stocks were subjected to whole-genome sequencing. In total, 943 isolates from the JARBB collection (437 from 2004, 270 from 2007, and 236 from 2010) were subjected to whole-genome sequencing.

The year and month of isolation, specimen type, IPD/non-IPD status, and region of isolation for each isolate are provided in Supplementary Table S1. Specimen type was categorized as sputum, throat swab, urine, feces, blood, or "other specimen," reflecting the level of detail recorded in the original source materials. No cerebrospinal fluid (CSF) samples were explicitly identified; however, the possibility that CSF isolates are included within the "other specimen" category cannot be excluded. For this reason, isolates categorized as "other specimen" were recorded with an IPD/non-IPD status of "unknown" in Supplementary Table S1.

Trimming and *de novo* assembly

Raw reads were quality-filtered and trimmed using fastp v0.23.4 [4]. Adapter sequences were automatically detected and removed, and polyX and polyG tails were trimmed. Reads <80% of the mean read length were discarded; all other parameters were set to their default values. The 80% mean read-length threshold was determined by calculating the mean read length from the first 100 reads using SeqKit v2.8.2 [5]. Trimmed reads from each isolate were assembled using Shovill v1.1.0 [6], and contigs <300 bp were discarded.

In silico serotyping

In silico serotyping was performed using SeroBA v1.0.2 [7] with default settings. When the serotype could not be determined because of low coverage, the isolate was reanalyzed using Pathogenwatch seroba-v2.0.4 [8].

Penicillin-binding protein (PBP) typing

When a PBP type was designated as "NEW," the corresponding isolates were further analyzed locally as described below. Nucleotide sequences of *pbp1a*, *pbp2b*, and *pbp2x* genes were extracted using ABRicate [9] and converted to amino acid sequences using EMBOSS v6.6.0 [10]. The resulting amino acid sequences were analyzed using BLASTp v2.15.0 [11, 12] against a reference database. In the present study, two databases were used: the SPN_Reference_DB database [13, 14] was used as the primary database, and when a PBP type was assigned as "NEW," the isolate was reanalyzed using a second database based on previous studies from Japan [15, 16].

Phylogenetic analysis using Gubbins

Trimmed reads were mapped to the complete reference genome of *Streptococcus pneumoniae* serotype 19A-ST2331 (GenBank accession number AP026921.1) to generate genome alignments. Read mapping was performed using Snippy v4.6.0 [17].

A recombination site-censored maximum-likelihood tree was constructed using Gubbins v3.3.4 [18]. IQ-TREE-FAST was used for the initial tree, and RAxML-NG was employed for subsequent tree building and model fitting, with up to 10 iterations performed. Bootstrapping was performed with 100 replicates. All other parameters were set to their default values.

Bayesian dating analysis using BEAST X

A recombination-censored maximum-likelihood phylogeny was generated using Gubbins v3.3.1 [18]. Temporal signal was assessed by permutation testing using BactDating v1.1.2 [19] based on the Gubbins output. After confirmation of a significant temporal signal, recombinant sites were masked with Ns in the core-genome alignment using maskrc-svg v0.5 [20] with the --gubbins option to generate a recombination-masked alignment for molecular dating analysis.

Bayesian molecular dating was then performed using BEAST X v10.5.0 [21]. Four combinations of clock models and tree priors were compared: a strict clock or an uncorrelated lognormal relaxed clock, each combined with either a coalescent constant-size prior or a coalescent Hamiltonian Monte Carlo SkyGrid prior. Model fit was evaluated using path sampling, and the best-fit model was selected for the final analysis. Markov chain Monte Carlo (MCMC) chains were run for 10^8 iterations, and convergence was assessed using Tracer v1.7.2 [22], with all key parameters showing effective sample size (ESS) values >200.

Supplementary Results

Amino acid sequence of PBP2x designated as a "NEW" type

>PBP2x_DPP04212A
MKWTKKVIRYATKNRKSPAENRRRVGKSLSLLSVFVFAVFLVNFAVIIGTGTRFGTDLAKEAKKVHQTTRTVPAKRGT
IYDRNGVPIAEDATSYNVYAVIDENYKSATGKILYVEKTQFNKVAEVFHKYLDMEESYVREQLSQPNLKQVSFGSKG
NGITYANMMAIKKELETAEVKGIDFTTSPNRSYPNGQFASSFIGLAQLHENEDGSKSLLGTSGLESSLNTILAGTDGII
TYEKDRVGNIVPGTEQVSQQTVDGKDVYTTISSTLQSFMETQMDAFLEKVKGKYMTATLVSAKTGEILATTQRPTF
NADTKEGITEDFVWRDILYQSNYEPGSGMKVMTLASSIDNNTFPSGEYFNSSEFKIADATTRDWDVNEGLTTGGM
MTFLQGFAHSSNVGMSLLEQKMGDATWLDYLKRFKFGVPTRFGLTDEYAGQLPADNIVSIAQSSFGQGISVTQTQ
MLRAFTAIANDGVMLEPKFISAIYDTNNQSVRKSQKEIVGNPVSKEAASTTRNHMILVGTDPLYGTMYNHYTGKPII
TVPGQNVAVKSGTAQIADEKNGGYLVGSTNYIFSVVTMNPAENPDFILYVTVQQPEHYSGIQLGEFATPILERASAM
KESLNLQSPAKNLDKVTTESSYAMPSIKDISPGELAEALRRNIVQPIVVGTGTKIKETSVEEGTNLAPNQQVLLLSDKV
EEIPDMYGWKKETAETFAKWLDIELEFEGSGSVVQKQDVRTNTAIKNIKKITLTLGD

*Red characters correspond to the region of the PBP2x type database.

Dating the serotype 19A-ST2331 subclade in Japan

To infer the timescale of diversification of the serotype 19A-ST2331 subclade in Japan, root-to-tip regression analysis using BactDating was performed, which showed a positive correlation between sampling time and genetic divergence (R^2 = 0.272; correlation coefficient = 0.522; Figure S3), supporting the presence of sufficient temporal signal for molecular dating analysis.

Among the four combinations of clock models and tree priors tested in BEAST, the uncorrelated lognormal relaxed clock model combined with a coalescent constant-population-size prior showed the best fit based on path sampling.

Supplementary Figures

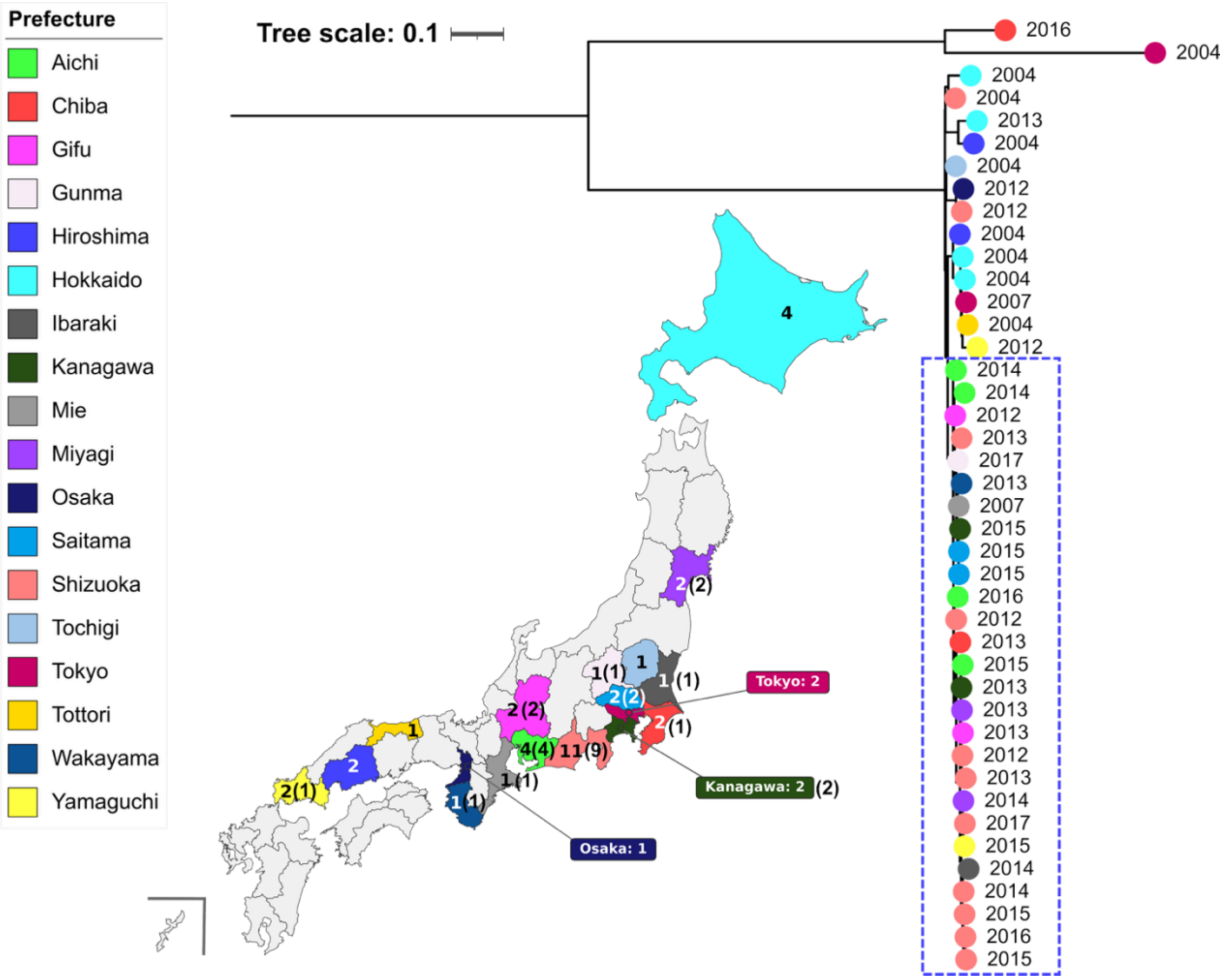

Figure S1. Maximum-likelihood phylogenetic tree of 42 CC2331 isolates collected in Japan, including serotype 19A-ST2331 (n = 34), serotype 19A-other STs (n = 6), serotype 9N-ST405 (n = 1), and serotype 12F-ST18693 (n = 1), constructed using kSNP4. Colored dots at the tips of the tree indicate the prefectures where the isolates were collected, with the numbers indicating the year of isolation. In the map of Japan, the same color codes and numbers used in the phylogenetic tree indicate the geographic locations of the corresponding prefectures and the number of isolates recovered from each prefecture. Numbers shown in parentheses indicate the number of isolates belonging to the *mefA/E*-positive serotype 19A-CC2331 subcluster (enclosed by the blue dashed line) recovered from each prefecture. The 27 isolates enclosed by the blue dashed line correspond to the *mefA/E*-positive serotype 19A-CC2331 subcluster shown in Figure 4. Isolates in this subcluster were recovered from 12 prefectures between 2007 and 2017. In this phylogenetic tree, isolates collected in 2004 and 2007 were derived from the JARBB (Japan Antimicrobial Resistant Bacterial Bank) collection, whereas those collected between 2012 and 2017 were derived from the Pneumocatch collection.

a.

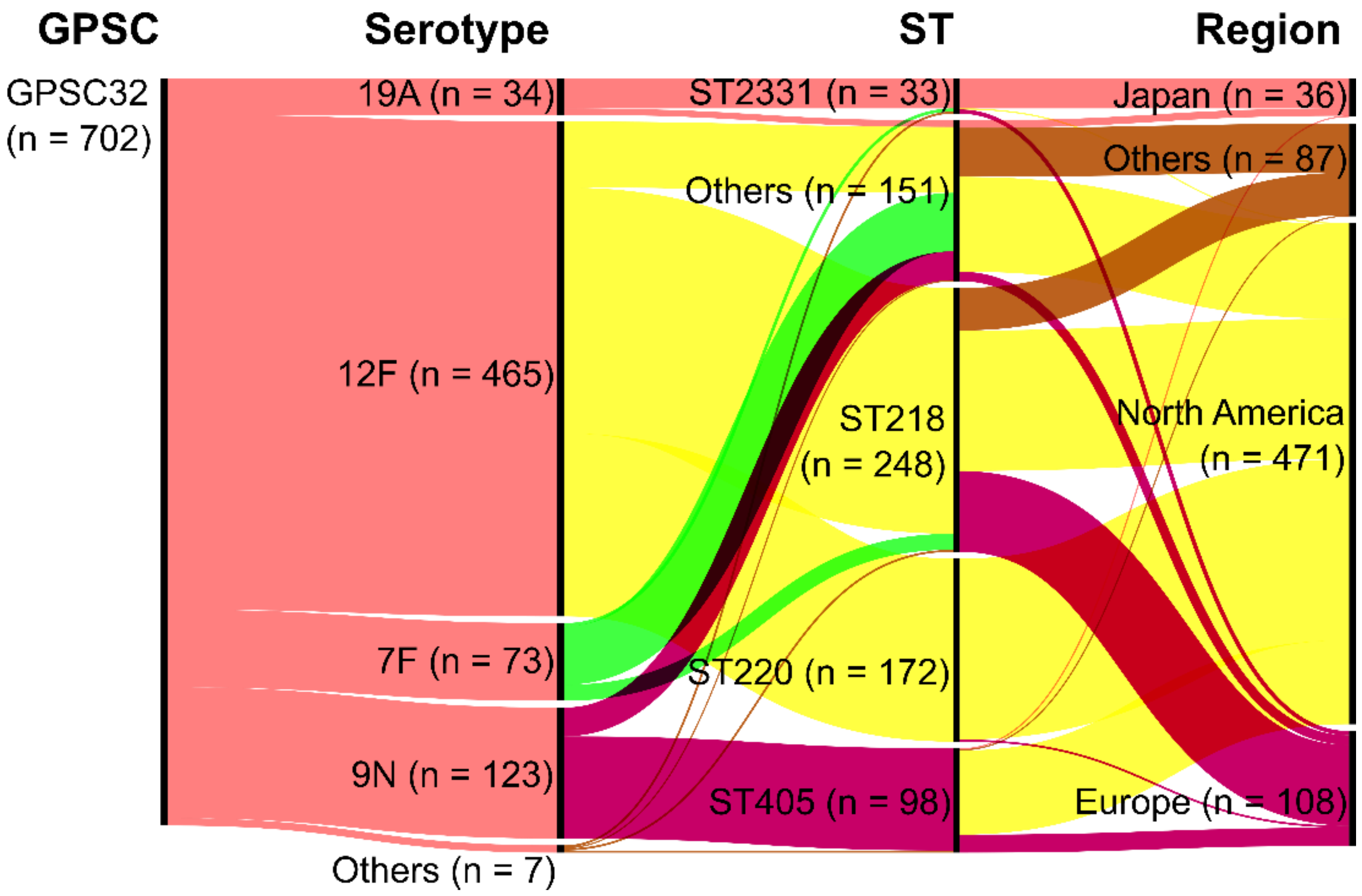
GPSC
Serotype
ST
Region
GPSC32
(n = 702)
19A (n = 34)
12F (n = 465)
7F (n = 73)
9N (n = 123)
Others (n = 7)
ST2331 (n = 33)
Others (n = 151)
ST218
(n = 248)
ST220 (n = 172)
ST405 (n = 98)
Japan (n = 36)
Others (n = 87)
North America
(n = 471)
Europe (n = 108)

b.

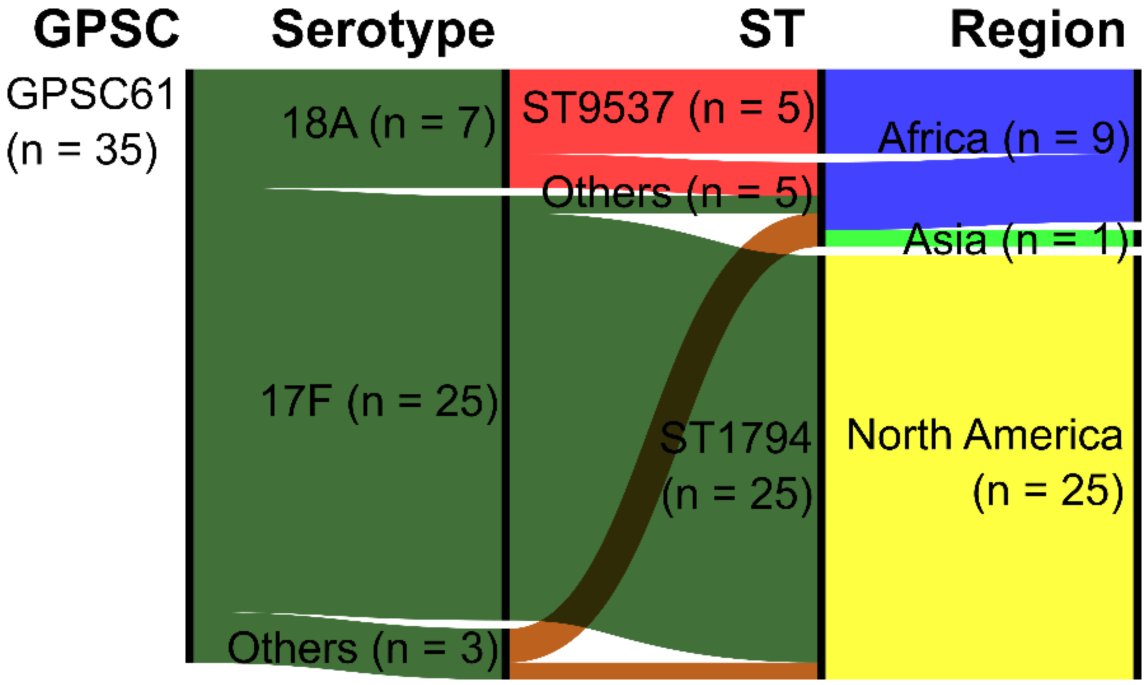
GPSC
Serotype
ST
Region
GPSC61
(n = 35)
18A (n = 7)
17F (n = 25)
Others (n = 3)
ST9537 (n = 5)
Others (n = 5)
ST1794
(n = 25)
Africa (n = 9)
Asia (n = 1)
North America
(n = 25)

c.

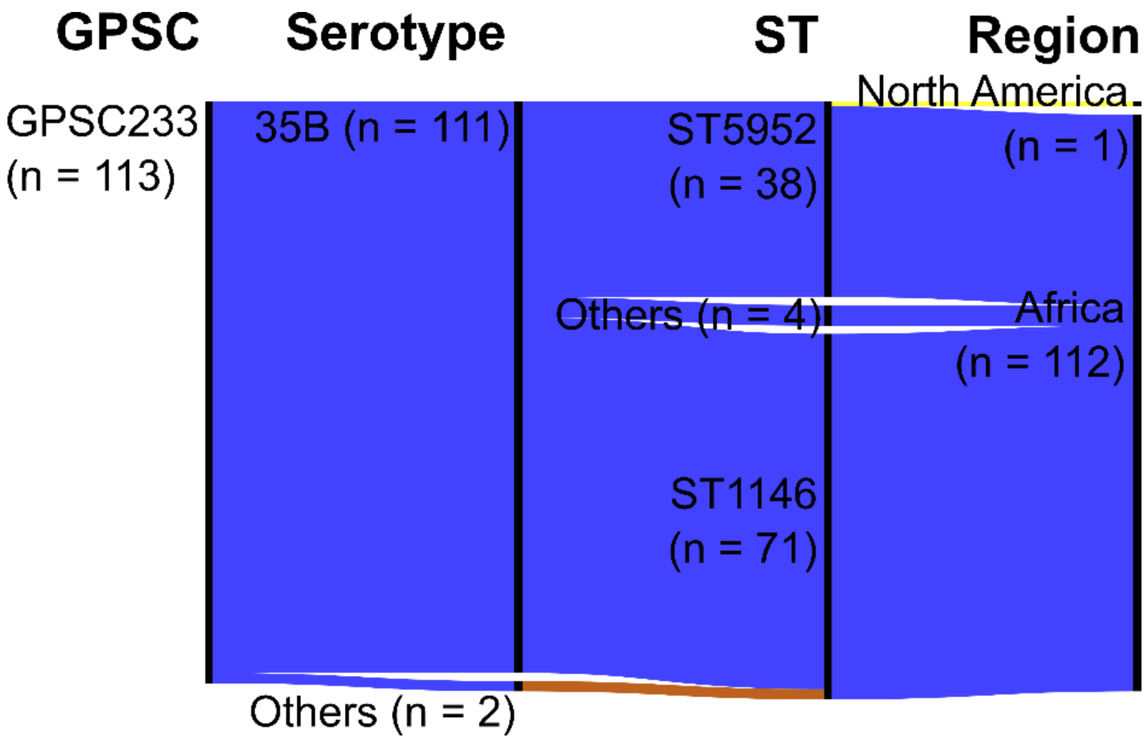

d.

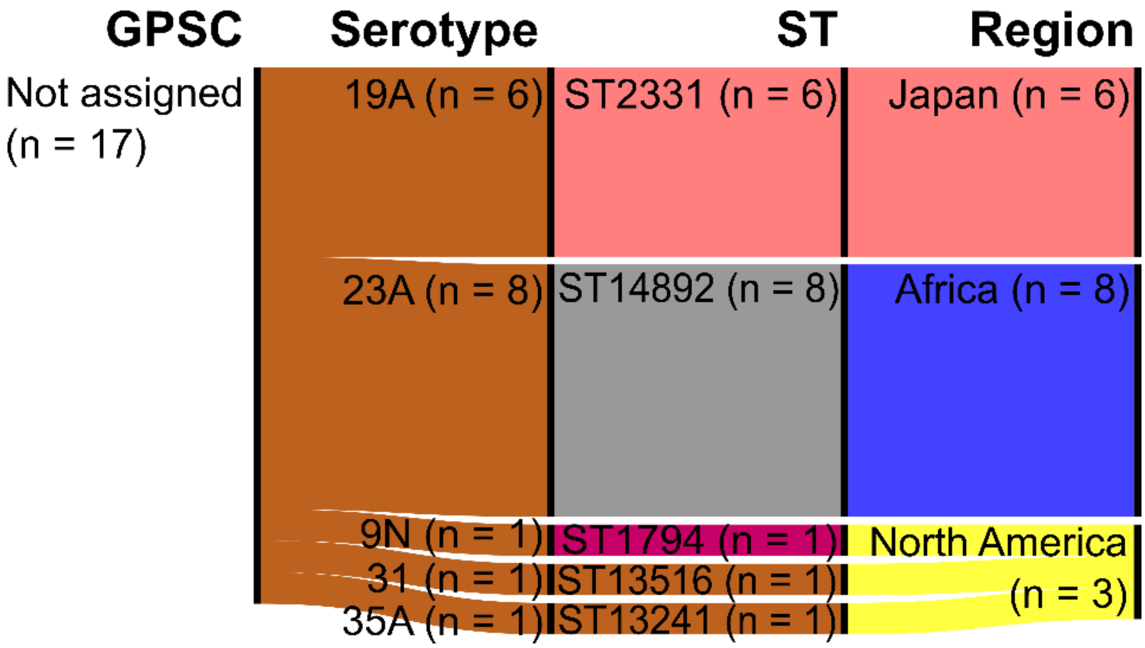

Figure S2. Relationships among GPSC, serotype, sequence type (ST), and region of isolation. Panels show (a) GPSC32, (b) GPSC61, (c) GPSC233, and (d) isolates not assigned to a GPSC. Numbers shown beneath each category represent the number of corresponding isolates.

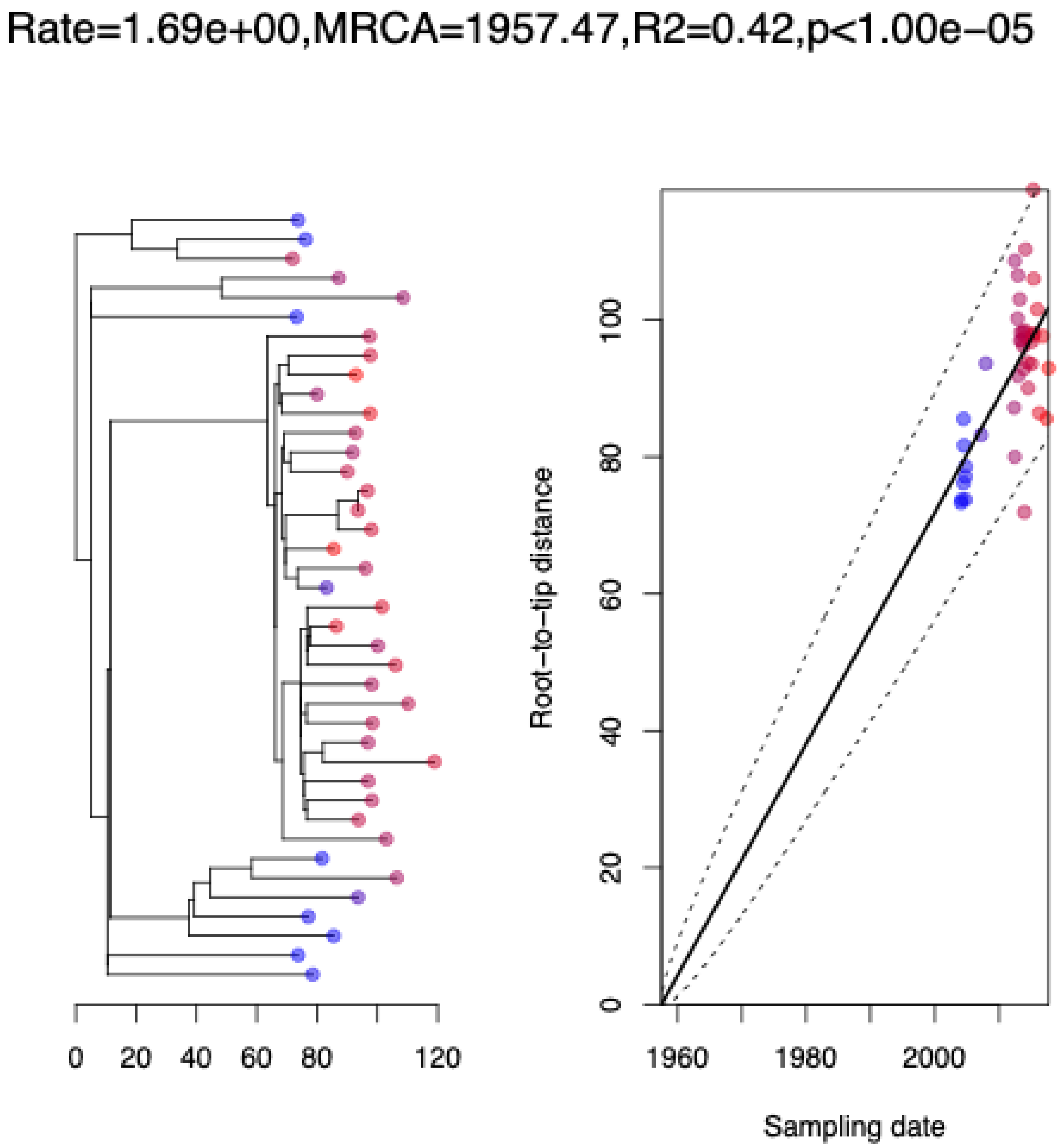

Figure S3. Assessment of temporal signal among 40 serotype 19A-CC2331 isolates from Japan using BactDating. Root-to-tip regression analysis and permutation testing demonstrated a significant temporal signal.

Supplementary References

1. **Tateda K, Ohno A, Ishii Y, Murakami H, Yamaguchi K et al.** Investigation of the susceptibility trends in Japan to fluoroquinolones and other antimicrobial agents in a nationwide collection of clinical isolates: A longitudinal analysis from 1994 to 2016. *J Infect Chemother* 2019;25(8):594-604.
2. **Nakano S, Fujisawa T, Ito Y, Chang B, Suga S et al.** Serotypes, antimicrobial susceptibility, and molecular epidemiology of invasive and non-invasive Streptococcus pneumoniae isolates in paediatric patients after the introduction of 13-valent conjugate vaccine in a nationwide surveillance study conducted in Japan in 2012-2014. *Vaccine* 2016;34(1):67-76.
3. **Nakano S, Fujisawa T, Ito Y, Chang B, Matsumura Y et al.** Nationwide surveillance of paediatric invasive and non-invasive pneumococcal disease in Japan after the introduction of the 13-valent conjugated vaccine, 2015-2017. *Vaccine* 2020;38(7):1818-1824.
4. **Chen S, Zhou Y, Chen Y, Gu J**. fastp: an ultra-fast all-in-one FASTQ preprocessor. *Bioinformatics* 2018;34(17):i884-i890.
5. **Shen W, Le S, Li Y, Hu F**. SeqKit: A Cross-Platform and Ultrafast Toolkit for FASTA/Q File Manipulation. *PLoS One* 2016;11(10):e0163962.
6. **Seemann T**. Shovill. Available from: https://github.com/tseemann/shovill.
7. **Epping L, van Tonder AJ, Gladstone RA, The Global Pneumococcal Sequencing Consortium, Bentley SD et al.** SeroBA: rapid high-throughput serotyping of Streptococcus pneumoniae from whole genome sequence data. *Microb Genom* 2018;4(7).
8. **Pathogenwatch**. A Global Platform for Genomic Surveillance. Available from: https://pathogen.watch/.
9. **Seemann T**. ABRicate. Available from: https://github.com/tseemann/abricate.
10. **Rice P, Longden I, Bleasby A**. EMBOSS: the European Molecular Biology Open Software Suite. *Trends Genet* 2000;16(6):276-277.
11. **Altschul SF, Gish W, Miller W, Myers EW, Lipman DJ**. Basic local alignment search tool. *J Mol Biol* 1990;215(3):403-410.
12. **Camacho C, Coulouris G, Avagyan V, Ma N, Papadopoulos J et al.** BLAST+: architecture and applications. *BMC Bioinformatics* 2009;10:421.
13. **Metcalf BJ, Gertz RE, Jr., Gladstone RA, Walker H, Sherwood LK et al.** Strain features and distributions in pneumococci from children with invasive disease before and after 13-valent conjugate vaccine implementation in the USA. *Clin Microbiol Infect* 2016;22(1):60 e69-60 e29.
14. **Metcalf BJ**. SPN_Reference_DB. Available from: https://github.com/BenJamesMetcalf/Spn_Scripts_Reference/tree/master/SPN_Reference_DB [accessed 29 May 2024].
15. **Nakano S, Fujisawa T, Ito Y, Chang B, Matsumura Y et al.** Penicillin-Binding Protein Typing, Antibiotic Resistance Gene Identification, and Molecular Phylogenetic Analysis of

Meropenem-Resistant Streptococcus pneumoniae Serotype 19A-CC3111 Strains in Japan. *Antimicrob Agents Chemother* 2019;63(9).

16. **Nakano S, Fujisawa T, Chang B, Ito Y, Akeda H et al.** Whole-Genome Analysis-Based Phylogeographic Investigation of Streptococcus pneumoniae Serotype 19A Sequence Type 320 Isolates in Japan. *Antimicrob Agents Chemother* 2022;66(2):e0139521.
17. **Seemann T**. Snippy. Available from: https://github.com/tseemann/snippy.
18. **Croucher NJ, Page AJ, Connor TR, Delaney AJ, Keane JA et al.** Rapid phylogenetic analysis of large samples of recombinant bacterial whole genome sequences using Gubbins. *Nucleic Acids Res* 2015;43(3):e15.
19. **Didelot X, Croucher NJ, Bentley SD, Harris SR, Wilson DJ**. Bayesian inference of ancestral dates on bacterial phylogenetic trees. *Nucleic Acids Res* 2018;46(22):e134.
20. **Kwong J, Seemann T**. maskrc-svg. Available from: https://github.com/kwongj/maskrc-svg.
21. **Baele G, Ji X, Hassler GW, McCrone JT, Shao Y et al.** BEAST X for Bayesian phylogenetic, phylogeographic and phylodynamic inference. *Nat Methods* 2025;22(8):1653-1656.
22. **Rambaut A, Drummond AJ, Xie D, Baele G, Suchard MA**. Posterior Summarization in Bayesian Phylogenetics Using Tracer 1.7. *Syst Biol* 2018;67(5):901-904.
